# A Practical Treatise on the Most Obvious Diseases Peculiar to Horses

**Published:** 1863-12

**Authors:** 


### Book Notices.

A Practical Treatise on the most Obvious Diseases Peculiar to Horses, Together with Directions for their most Rational Treatment; Containing, also, some Valuable Information on the Art of Shoeing Horses. By George H. Dadd, V.S., Author of "Anatomy and Physiology of the Horse," "Modern Horse Doctor," etc., etc., and Principal of the Veterinary School of Chicago. Chicago: S. C. Griggs & Co. New York: Blakeman & Mason; C. M. Saxton. 1863.

We are pleased, in looking over this work, to see the steady advance Veterinary Medicine is making. Idle notions of disease are being set aside, and the Veterinary Surgeon, calling to his aid scientific modes of investigation, and acting in the light of facts in human pathology and therapeutics, is rapidly placing his work on a plane parallel with ours.

We commend the book to all interested in horses; and the physician may find in it some useful hints. For instance: one of Dr. Dadd's patients was a "regular hysterical subject during the menstrual periods, uncontrollable and amaruotic;" the diseased ovaries are removed, and a permanent cure follows. Here is material for the pathologist and therapeutist, and especially for the psychonosologist.

